# The targeting mechanism of DHA ligand and its conjugate with Gemcitabine for the enhanced tumor therapy

**DOI:** 10.18632/oncotarget.1969

**Published:** 2014-05-13

**Authors:** Siwen Li, Jingyi Qin, Caiping Tian, Jie Cao, Guissi Fida, Zhaohui Wang, Haiyan Chen, Zhiyu Qian, Wei R Chen, Yueqing Gu

**Affiliations:** ^1^ Department of Biomedical Engineering, State Key Laboratory of Natural Medicines, School of Life Science and Technology, China Pharmaceutical University; ^2^ Department of Biomedical Engineering, School of Automation, Nanjing University of Aeronautics and Astronautics, Nanjing, China; ^3^ Department of Engineering and Physics, University of Central Oklahoma, Edmond, Oklahoma

**Keywords:** Docosahexaenoic acid, Near-infrared imaging, Tumor targeting, Phosphatidylethanolamine, Gemcitabine, tumor therapy

## Abstract

Docosahexaenoic acid (DHA), an omega-3 C22 natural fatty acid serving as a precursor for metabolic and biochemical pathways, was reported as a targeting ligand of anticancer drugs. However, its tumor targeting ability and mechanism has not been claimed. Here we hypothesized that the uptake of DHA by tumor cells is related to the phosphatidylethanolamine (PE) contents in cell membranes. Thus, in this manuscript, the tumor-targeting ability of DHA was initially demonstrated *in vitro* and *in vivo* on different tumor cell lines by labeling DHA with fluorescence dyes. Subsequently, the tumor targeting ability was then correlated with the contents of PE in cell membranes to study the uptake mechanism. Further, DHA was conjugated with anticancer drug gemcitabine (DHA-GEM) for targeted tumor therapy. Our results demonstrated that DHA exhibited high tumor targeting ability and PE is the main mediator, which confirmed our hypothesis. The DHA-GEM displayed enhanced therapeutic efficacy than that of GEM itself, indicating that DHA is a promising ligand for tumor targeted therapy.

## INTRODUCTION

Cancer has been known as the second leading cause of death in the world due to the late diagnosis and lack of the specificity of most chemotherapeutic drugs [1-2]. Thus earlier diagnosis and targeted therapy are the main focus of the researchers nowadays. The tumor targeting ligands play a crucial role in the development of tumor contrast agents and targeted anticancer drugs. [3-10]

Docosahexaenoicacid (DHA), an omega-3 C22 natural fatty acid with six cis double bonds, is a precursor for metabolic and biochemical pathways. Studies have circumstantiated that DHA has the activity of inhibiting several kinds of cancer cells. Various mechanisms of its anticancer activity were proposed such as modulating the cellular proliferation, apoptosis and differentiation, increasing drug transportation across the tumor cell membrane, generating free oxygen radicals and lipid peroxidation [11-18]. Due to the increased tumor membrane transportation and its relatively anticancer activity DHA has been conjugated with anti-tumor drugs to increase therapeutic efficacies. For example, DHA-paclitaxel, which is currently under phase 3 clinical study, has showed less toxicity, higher tumor accumulation concentration, higher anti-tumor efficacy compared to the paclitaxel [19-26]. DHA-Doxorubicin is significantly more efficacious than free Dox, with an increased uptake of DHA-Dox by tumors and an increased half-life in the body [27].

However, the reason for the high concentration of DHA in tumor cells was not clearly elucidated. Teague et al reported that a DHA-fluorescent probe was highly uptaken by EL4 cells at 37°C than at 23°C, which could be attributed to the high fluidity of the cell membrane at 37°C, while it is more ordered at 23°C [28]. Robinson et al found that DHA was initially incorporated into phosphatidylethanolamine (PE) accompanied by a less amounts into phosphatidylcholine (PC) or other phospholipid classes [29-30]. Liu et al found that PE had higher expression in gastric cancer cells than that in the normal gastric mucosa [31].Based on these reports, we hereby hypothesize that the high accumulation of DHA in tumor cells is directly related with the PE contents in cell membrane. As Christopher Stubbs reported that PE and other phospholipid had a positive correlation to the fluidity of cell membrane [30]. The amount of PE could be reflected by the fluidity of cell membrane. In this study, we will measure the fluidity of cell membrane to investigate the correlation of tumor targeting ability of DHA with the PE contents in cell membrane.

Optical imaging has been gaining a lot of interest, especially near infrared (NIR) fluorescence imaging for its obvious advantages as a non-invasive technique for *in vivo* real time monitoring or tracing biological information and signals in small animals. In this study, a near infrared dye Cypate will be conjugated to DHA to form a fluorescence probe for investigating the tumor targeting ability of DHA in different tumor lines.

Gemcitabine (GEM) hydrochloride (HCl) is approved for the treatment of a wide spectrum of solid tumors. But its use is limited by its short half-life, toxic and side effects as well as drug resistance [32]. The modification of GEM with DHA may improve the tumor targeting ability and the anticancer efficacy. Thus, we will synthesize a brand new antitumor prodrug by covalently conjugating DHA with gemcitabine to strengthen its anti-tumor activity and reduce the drug dosage to reduce its toxicity.

## RESULTS AND DISCUSSION

### Synthesis and characterization of DHA-Cypate probe

**Figure 1:**
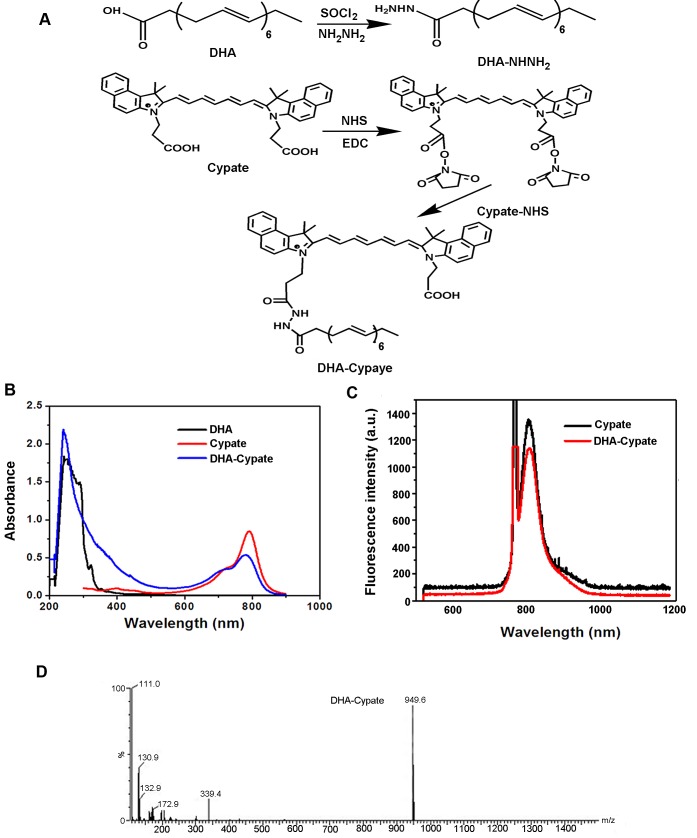
A, synthetic scheme of DHA-Cypate, through modification of the carboxy group of DHA and Cypate using NHNH as reaction linkage B, the absorption spectra of DHA-Cypate, free DHA, free Cypate. C, fluorescence emission spectra of DHA-Cypate, free Cypate. D, mass spectrum of DHA-Cypate.

The procedure of synthesis for DHA-Cypate is shown in Fig. 1 A. The amino-group of the modified DHA was reacted with the NHS ester of Cypate. In the process, the intermediate product containing -COCl group was obtained from the chemical reaction between SOCl_2_ and –COOH. Then, the reaction between -COCl group and NH_2_NH_2_ resulted in the intermediate product (amino modified DHA). In order to enhance the reaction efficiency, excess SOCl_2_ and NH_2_NH_2_ were used to allow sufficient reactions. The reaction of amino modified DHA and Cypate was carried out after the intermediate product was verified by MS analysis. NHS and EDCI served as catalysts in this reaction with a molar ratio to Cypate of 3:3:1. To improve the reaction efficacy, the catalysis system and feed ratios of different components were optimized and verified by the step-by-step Tcl evaluation. The crude product DHA-Cypate was subsequently purified by silica gel column. The purified product was characterized, as shown in Fig. 1. The maximum absorption wavelengths of DHA-Cypate were matched exactly with that of free DHA and Cypate at 270 nm and 780 nm respectively, indicating the successful conjugation of DHA and Cypate (Fig. 1B). Fluorescence spectra in Fig. 1C exhibited the same fluorescence peak of DHA-Cypate (red) and Cypate (black) at 810 nm, implying that the conjugation of DHA with Cypate did not affect the fluorescence of Cypate. The MS analysis in Fig. 1D displayed that the molecular weight of DHA-Cypate is 949.6, the sum of DHA (328.5), Cypate (625), NH_2_NH_2._HCL(70) subtracting two molecular H_2_O (32), further confirm the successful conjugation of DHA-Cypate.

### *In vitro* cell targeting and uptake mechanism of DHA based probes

### Cell targeting by DHA-based probes

**Figure 2:**
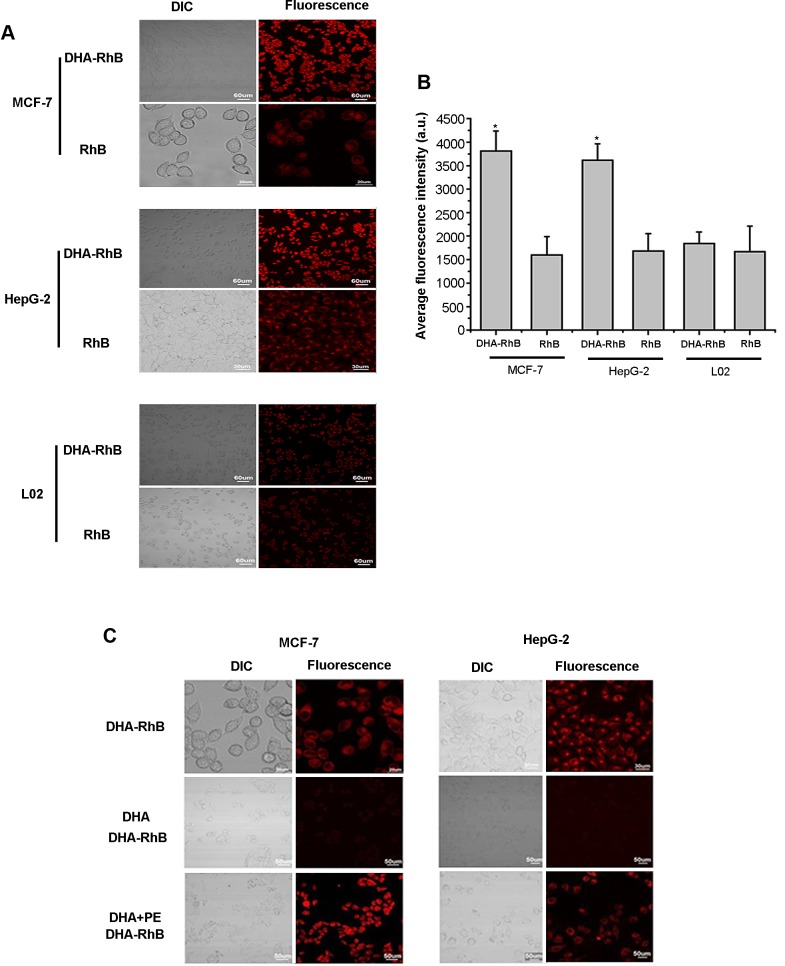
A, laser confocal fluorescence microscopy images of MCF-7, HepG-2, L02 cells incubated with DHA-RhB and free RhB B, mean fluorescence intensity of MCF-7, HepG-2, L02 cells incubated with DHA-RhB and free RhB. C, laser confocal fluorescence microscopy images of MCF-7, HepG-2 cells incubated with DHA-RhB, DHA block DHA-RhB, DHA combined with PE recover the DHA-RhB. Data are given as mean ± SD (n=5). *, *P*<0.05. DIC, differential interference contrast.

Tumor cellular uptake of DHA-based probes was determined by confocal microscopy. Visible fluorescence dye RhB was used to replace Cypate in the probe. As shown in Fig. 2A, the fluorescence in the cells incubated with the DHA based probe displayed higher intensity than that of free RhB itself in all the cell lines. More importantly, the fluorescence in MCF-7 cells incubated with DHA-RhB exhibited brighter fluorescence (means higher emission intensity) than that of HepG-2 cells. The normal cells L02 displayed weakest fluorescence signal even incubated with DHA-RhB. The quantitative analysis was plotted in Fig. 2B. The fluorescence intensity decreased in the order of MCF7>HepG2>L02. These results indicated that DHA-based probe has higher uptake by tumor cells, with better targeting ability in MCF-7 cells than that of HepG-2 cells.

### Investigation of the uptake mechanism

To further study the tumor targeting mechanism of DHA, we designed an *in vitro* DHA blocking and PE competition binding experiment. As shown in Fig. 2C, cells incubated with DHA-RhB and cells in PE competition binding group both had stronger intracellular fluorescence intensity than that of the cells in DHA blocking group. DHA-RhB was rarely up-taken by the cells in the DHA blocking group, while in the PE competition binding group, DHA-RhB was highly presented intracellular. The blocking experiments indicated that the binding of DHA with PE plays key role in the cellular uptake. PE is one of the components within the phospholipid bilayer of cell membranes. When free DHA was incubated with the cells, the DHA was preferentially bound with PE to form DHA-PE compound and entered the cells afterwards. Therefore, as a large number of PE was initially occupied by free DHA, the added DHA-RhB probe would be blocked outside the cell membrane, resulting in the low cellular uptake (Fig. 2C). In contrast, in the PE competition binding DHA group, free DHA and free PE were added simultaneously into the medium prior to DHA-PE binding, allowing the PE in the cell membrane remained unoccupied. Thus, the added DHA-RhB had the chance to combine with unoccupied PE in the cell membranes and enter the cells in large quantity (Fig.2C). All the results indicated that PE contents in the cellular membrane was a major component for the cellular uptake of DHA.

### The fluidity of cell membrane

**Figure 3:**
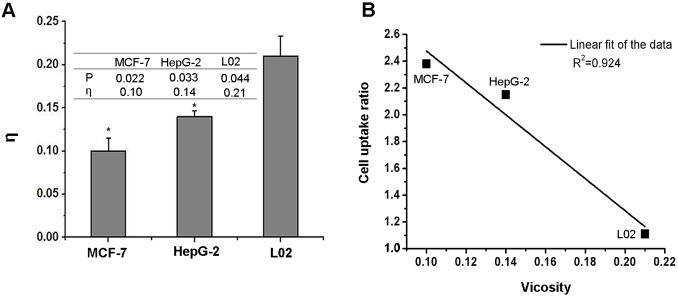
A, the value of the fluorescent polarization P and the viscosity η in MCF-7, HepG-2, L02 cells B, linear fit of the value of cell uptake ratio and viscosity, R^2^=0.924. Data are given as mean ± SD (n=5). *, *P*<0.05.

To confirm our hypothesis, PE contents in the cell membranes of different cell lines were determined. Since the exact measurement of PE amount in cell membrane is very difficult, we determined the amount of PE based on the previous reports that a positive correlation between the fluidity of the cell membrane and the PE within phospholipids existed [30]. Fluidity itself is inversely proportion to the viscosity which can be easily measured. Tumor cells (MCF-7 and HepG2) and normal liver cells (L02) were used for the fluidity measurements. As shown in Fig. 3A, the fluidity deduced from the measured fluorescence polarization is in the order of MCF-7>HepG-2>L02, implying that the amount of PE within the cellular membranes follows the same order, MCF-7>HepG-2>L02. The membrane fluidity of L02 cells was much lower than that of the tumor cells. In addition, HepG-2 cells had a lower fluidity compared to MCF-7cells. In our previous cellular uptake study (Fig. 2A-2B), DHA-based probe had a higher accumulation in MCF-7 than that of HepG-2. Weak signal was observed in the normal L02 cells. The cellular uptake of DHA-based probe displayed close relationship with the PE contents. To correlate the targeting ability of DHA-based probe with the PE contents in tumor cell membrane, the cell uptake ratios was plotted against viscosity in Fig. 3B. A linear correlation (R^2^=0.924) between cell uptake and T/N and viscosity was obtained, which further confirmed our hypothesis. Above results support our proposed mechanism that DHA entering cells is related to the PE contents in the cell membranes. As tumor cells have higher PE contents, DHA based probe exhibits better tumor targeting ability.

### Cytotoxicity of DHA based probes

MCF-7 breast tumor cell line, HepG-2 liver tumor cell line and L02 normal liver cell line were treated with different concentrations of DHA-based probe(Cypate)(from 1.0 to 20 nM/L) for 24 h, and then cell viability was evaluated by MTT assay (Fig. supplementary 1). The tumor cells treated with DHA-Cypate have not exhibited distinct anti-proliferative activities as its concentration increased. However the cell viability of HepG-2 cells after incubation with DHA-Cypate was clearly higher than MCF-7 cells. This phenomenon may be correlated with the quantity difference of PE expression in different cell lines. It also predicts tumor killing effect of DHA in some cells along with its targeting activity.

### Tumor targeting ability in tumor bearing mice

**Figure 4:**
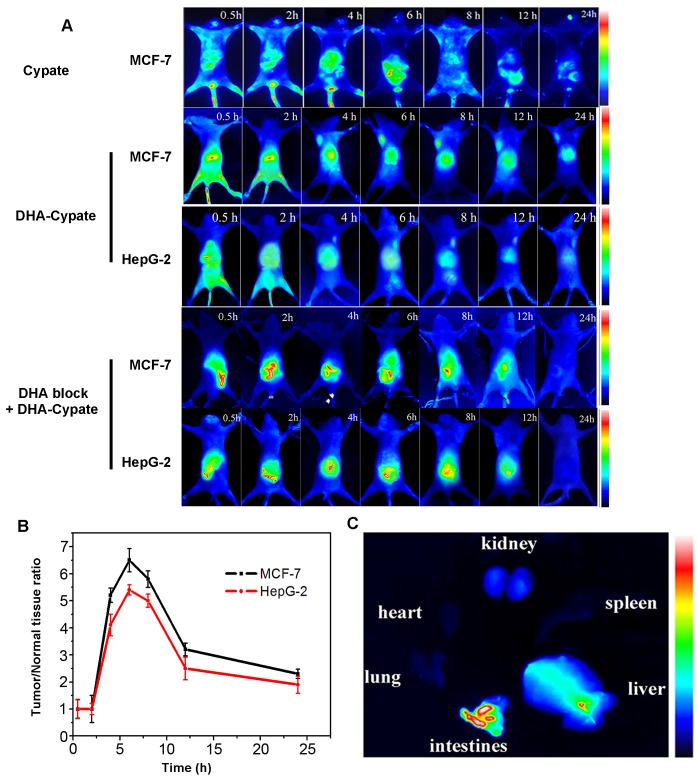
A, dynamics of Cyptae in MCF-7 bearing-nude mice, dynamics and tumor-targeting ability of DHA-Cypate in MCF-7, HepG-2 bearing nude mice, and DHA block DHA-Cypate in MCF-7, HepG-2 bearing nude mice B, tumor/normal tissue ratio (T/N ratio=[tumor signal -background signal]/[normal signal (muscle)-background signal]×100%) calculated from the ROIs at 2, 4, 6, 8, 12, 24hour postinjection of DHA-Cypate into MCF-7, HepG-2 bearing nude mice. C, NIR images of main organs excised from normal nude mice after intravenous injection of DHA-Cypate at 24h.

In order to evaluate the *in vivo* tumor-targeting ability of the DHA based probe, the near infrared dye, Cypate, was labeled with (or linked to) DHA to form DHA-Cypate probe. MCF-7 and HepG-2 tumor-bearing mice tail vein injected with the probe were monitored by NIR fluorescence imaging system at different time intervals (0.5 h, 2 h, 4 h, 6 h, 8 h, 12 h and 24 h). As shown in Fig. 4A, Cypate itself did not exhibit the tumor targeting ability. It spread over all the body at 0.5 h postinjection and gradually enters the liver and clear through the enterohepatic metabolism pathway after 24 hours circulation. Since Cypate and DHA share the same hydrophobic nature, the DHA-Cypate conjugate follows hepatic and intestinal metabolism, with initial biodistribution overall the body and slowly cleared from liver and intestines. The ex vivo imaging of main organs in Fig.4C confirmed the enterohepatic pathway, with bright fluorescence signals in liver and intestine. It indicated that when the probe was administrated into the blood stream, it would firstly arrive at liver by the blood circulation and then transferred to the intestine through bile duct, and gradually clear out of the body through the feces. The fluorescence was indeed observed in the feces (data did not show here). Weak fluorescence was observed in kidney DHA-Cypate showed longer circulation time in mice, which could be attributed to the stronger hydrophobicity of DHA-Cypate compared to Cypate alone. Most importantly, 4 hours post injection of DHA-Cypate, the tumor sites were obviously identifiable in both MCF-7 and HepG-2 bearing mice. The fluorescence intensity in tumor sites reached maxima at about 6 h post injection and maintained up to 24 h and then slowly faded away. Further, the fluorescence signals in MCF-7 tumor site were much brighter than those in HepG-2 tumor site at all the time points. Tumor to normal tissue contrast ratios were quantified and plotted in Fig. 4B. As shown, the ratio maximized in 6 h for both MCF-7 and HepG-2 mice models. The maximal T/N ratio of MCF-7 mice model (T/N = 6.5) was obviously higher than that of HepG-2 mice model (T/N = 5.3), which is consistent with the results from *in vitro* cellular uptake (Fig. 2B). Our results showed that DHA-based probe had excellent ability to target tumor cells of high PE contents.

To furtherstudy the targeting mechanism of DHA, NIR fluorescence images of the DHA blocked group and PE competition binding group were obtained. For DHA blocked group, no obvious fluorescence signal was observed in the tumor sites of the MCF-7 and HepG-2 tumor-bearing mice (Fig. 4A), while strong fluorescence signal was found in the PE recovered group (the signal intensity was same with DHA-Cypate group, data not shown).

The results of *in vivo* DHA blocking and PE competition binding experiments were consistent with that of the *in vitro* blocking experiment, reconfirming the role of PE in DHA uptake by tumor cells.

### Antitumor efficacy of DHA-GEM

### Synthesis and characterization of DHA-GEM

Severe toxic side effects and short half-life time are the main defects limiting the clinical application of gemcitabine. Targeted delivery and hydrophobic modification of gemcitabine may reduce the side effects and prolong its circulation for enhanced accumulation in the tumor. DHA was covalently conjugated to the inactive group N4-position of gemcitabine to increase its targeting activity, thus enhancing the drug efficacy and reducing the adverse effect.

**Figure 5:**
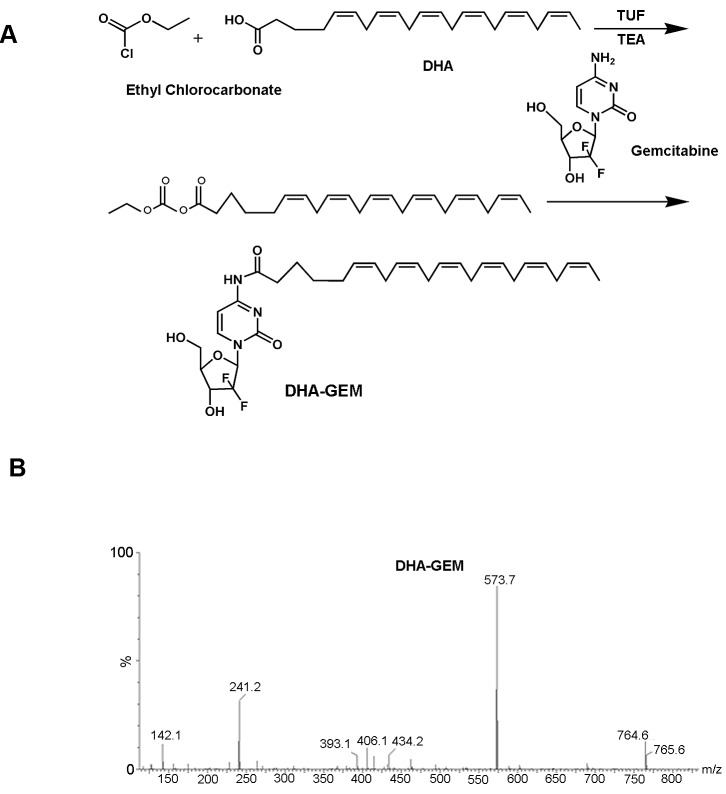
A, synthetic scheme of DHA-GEM, through modification of the carboxy group of DHA with the the primary amine group of gemcitabine B, mass spectrum of DHA-GEM.

The synthetic scheme for DHA-GEM is shown in Fig. 5A. In the reaction system, ethylchlorocarbonate played an important role similar to a catalyst and THF altered the pH, ensuring the continuity of the reaction and boosting the speed and efficacy. The relatively pure target compound DHA-GEM was obtained with silica gel column chromatography.

The molecular weight of DHA-GEM characterized by mass spectrometry is 573.7 (Fig. 5B), which is the sum of one DHA (328.5) and one gemcitabine (263.2), subtracting one water (18). The MS spectrum demonstrated the successful synthesis of DHA-GEM.

### *In vitro* antitumor activity of DHA-GEM

**Figure 6:**
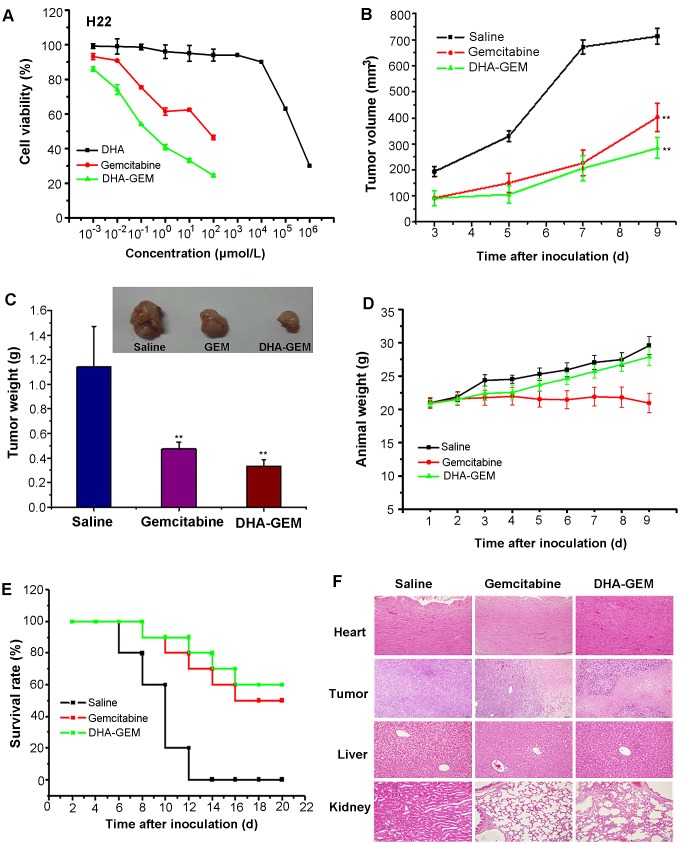
*In vitro* and vivo antitumor efficacy of DHA-GEM on H22 cells and H22 tumor-bearing mice A, Cell viability of H22 cells incubated with DHA-GEM and free gemcitabine. B, tumor volume of mice-bearing H22 tumors under different treatments (saline, free gemcitabine, or DHA-GEM, n=10/group). C, tumor weight and tumor picture of mice-bearing H22 tumors in different groups(saline, free gemcitabine, or DHA-GEM) on the 9th day after injection. D, body weights of mice bearing H22 tumors in different groups. E, the 20-day survival rates of mice after administration of saline, free gemcitabine, or DHA-GEM. F, hematoxylin and eosin-stained hearts, tumors, livers and kidneys of saline treated mice, gemcitabine-treated mice,or DHA-GEM–treated mice. Data are given as mean ± SD (n=10). ^**^, *P*<0.01.

**Figure 7:**
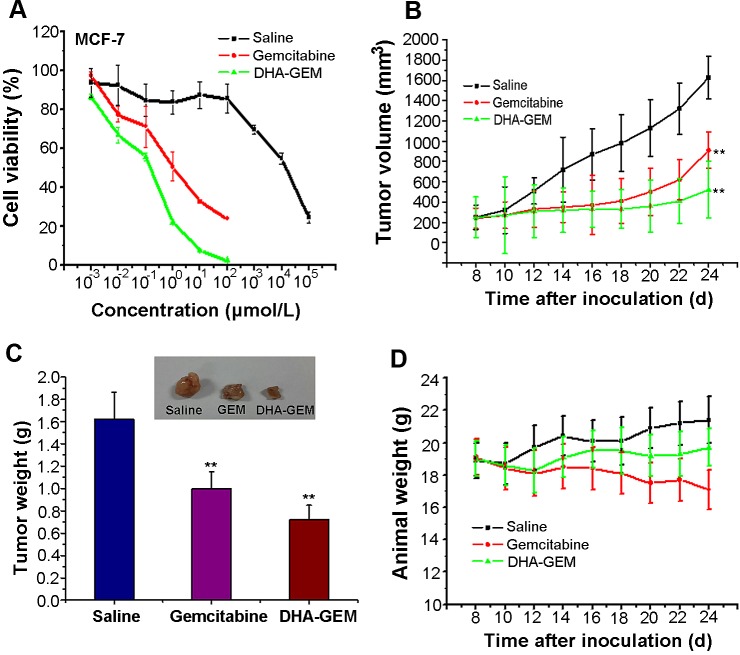
*In vitro* and vivo antitumor efficacy of DHA-GEM on MCF-7 cells and MCF-7 tumor-bearing nude mice A, Cell viability of MCF-7 cells incubated with DHA-GEM, free DHA and free gemcitabine. B, tumor volume of mice-bearing MCF-7 tumors under different treatments (saline, free gemcitabine, or DHA-GEM, n=6/group). C, tumor weights and tumor picture of mice-bearing MCF-7 tumors in different groups (saline, free gemcitabine, or DHA-GEM) on the 24th day after injection. D, body weights of mice bearing MCF-7 tumors in different groups. Data are given as mean ± SD (n=6). ^**^, *P*<0.01.

The *in vitro* cell experiments were conducted to determine the therapeutic effect of DHA-GEM in different tumor cell lines, as shown in Fig. supplementary 2. For comparison, the toxicity of DHA itself was investigated using MCF-7, HepG-2 and H22 cells (Fig. 7A, Fig. supplementary 2, Fig. 6A). The results showed that DHA within the dosage range of 0-100 μmol/L had no identifiable cytotoxicity on these tumor cells. However, when the amount of DHA reached 100 μmol/L, obvious cytotoxic effects were observed. Thus, the dosage of DHA was designed within the nontoxicity range (<100μmol/L) for all other *in vitro* experiments. Compared to gemcitabine alone, DHA-GEM displayed better therapeutic efficacy in all the tumor cells, including MCF-7(Fig. 6A), H22(Fig. 7A), HepG-2, A549, 7402, MDA- MB-231, 7901 (Fig. supplementary 2), demonstrating an impressive killing ability against tumor cells. Within the therapeutic window, more DHA-GEM (compare with gemcitabine) was uptaken by tumor cell, rendering high drug concentration. As a consequence, the toxic effects of GEM on normal cells could be reduced due to its reduced dosage in DHA-GEM. This result was consistent with our *in vitro* and *in vivo* targeting results, demonstrating that DHA was a promising ligand for tumor targeted therapy.

### *In vivo* antitumor therapeutic efficacy of DHA-GEM

*In vivo* antitumor efficacy of DHA-GEM was evaluated on H22 tumors -bearing Kunming mice and MCF-7 tumor-bearing nude mice. In the Kunming mice group, as shown in Fig. 6B, the tumors in the control group grew faster than that in the free gemcitabine and DHA-GEM treated mice. As expected, the tumor volume in DHA-GEM treated group showed less increase that of gemcitabine group. Finally, the tumor tissues at 9 days post injection of different treatments were exercised and weighted in Fig. 6C, with tumor inhibition rate of 70.1% for DHA-GEM and 58.6% for free gemcitabine. Obviously, DHA-GEM possessed higher therapeutic effect than that of free gemcitabine.

To compare the systemic toxicity, the body weight of subjected Kunming mice was recorded every other day and plotted in Fig. 6D. Steady but small weight increase could be observed in the mice of Saline and DHA-GEM groups, indicating the nontoxicity of DHA-GEM. The 20-day survival rates of mice-bearing H22 tumors in the DHA-GEM and free gemcitabine groups were 60% and 50%, respectively, whereas in the control group all the mice were dead on the 14th day (Fig. 6E).

After nine days administration, main organs (heart, liver and kidney) from the three groups (5/group) were collected for histological examination(Fig. 6F).The major organs, were observed in gemcitabine group and DHA-GEM group. In the liver group, we found that it had no obvious pathological changes both in the saline and gemcitabine groups, but a little in the DHA-GEM group. It is because that the modification with DHA has changed the metabolism of gemcitabine and has a slightly damage to the liver. In the kidney group, both gemcitabine and DHA-GEM groups have a little pathological damage. However the gemcitabine group is more seriously than the DHA-GEM group. It confirmed that conjugation with DHA could reduce the kidney toxicity of gemcitabine and decrease the side effects. For the hearts, no pathological changes occurred in both DHA-GEM group and the controlled group. On the contrary, pathological changes were observed in free gemcitabine group. While examination of tumor tissues in DHA-GEM group revealed pronounced pathological changes. Similarly, the antitumor efficacy of DHA-GEM was also investigated on the MCF-7 tumor bearing nude mice. As shown in Fig. 7B, the tumor volume indicated that DHA-GEM exhibited high tumor inhibition ratio than gemcitabine. By the same way, in the nude mice group, the tumor weight at 25th day was plotted in Fig. 7C, with tumor inhibition rate of 55.6% for DHA-GEM and 38.3% for free gemcitabine suggesting that the antitumor efficacy of DHA-GEM is better than free gemcitabine again. The body weight of subjected nude mice were plotted in Fig. 7D, also indicating the nontoxicity of DHA-GEM. It was concluded from above data that DHA-GEM demonstrated lower toxicity in normal organs and higher tumor killing ability than those of gemcitabine, though there were no obvious survival rate differences between the two groups.

## MATERIALS AND METHODS

### Materials and Instruments

Docosahexaenoic acid (DHA), Thionyl chloride (SOCl_2_), Pyridine (C_5_H_5_N), Hydrazine dihydrochloride (N_2_H_4_·2HCl), N-hydroxysuccinimide (NHS), N-(3-Dimethylaminopropyl)-N'- ethylcarbodiimide hydrochloride (EDCI), phosphatidylethanolamine (PE), Dimethyl sulfoxide (DMSO), 1,6-Diphenyl-1,3,5-hexatriene (DPH),triethylamine(TEA), tetrahydrofuran(THF), ethylchlorocarbonate,dimethylformamide(DMF), were purchased from Sigma-Aldrich (Shanghai, China). Hydrophobic NIR dye Cypate (MW:625) was prepared in our laboratory. All other reagents used in the study were analytical reagent grade (Shanghai Chemical Reagent Company, Shanghai, China) and used directly.

UV-Vis Spectrophotometer (JH 754PC, Shanghai, China) was used for the absorption measurements. S2000 eight-channel optical fiber spectrographotometer (Ocean Optics Corporation, USA) coupled with a NL-FC-2.0-763 semiconductor laser (λ=765.9 nm, Enlight, China) light was utilized for fluorescence spectra detection. Laser confocal fluorescence microscopy (FluoView™ FV1000, Olympus, Japan) was used for cell imaging. Mass spectrometry (MS,Q-TOF Micro,Water Company USA) was utilized to analyze the molecular weight of the product. PHS-25 pH meter (Shanghai, China) was used to measure the pH. Products were purified and identified by using silica gel column and Q-TOF Micro Mass Spectrometer (Waters, USA), respectively.

A NIR fluorescence imaging system was used to determine the distribution of the probes in mice. This home-built imaging system was reported in our previous works [33-34]. Briefly, the NIR system contains an excitation laser (λ=765.9 nm, NL-FC-2.0-763 laser light), a high sensitivity NIR CCD camera (PIXIS 512B, Princeton Instrumentation) and an 800 nm long pass filter for capturing the fluorescence emission from the tissue. In addition, another HLU32F400 808 nm laser (LIMO, Dortmund, Germany) was incorporated as background light to obtain the animal profile.

### Synthesis and characterization of DHA based near infrared fluorescent probes

#### Synthesis of DHA(-CONHNH_2_)

DHA (4.0 mg) and pyridine (50 μL) were added to SOCl_2_ solution (15 mL). The solution was heated slowly to 70°C with aqueous refluxing and stirred vigorously for two hours until there was no gas escape, and then pressure was reduced to remove remaining SOCl_2_. The intermediate product DHA(-COCl) was obtained. NH_2_NH_2_ (200 mg) and DHA(-COCl) were added to DMSO solution (20 ml) and stirred at 0°C overnight to obtain DHA(-CONHNH_2_).

#### Synthesis and characterization of DHA-Cypate

Cypate (10 mg) was firstly reacted with EDC·HCl (9.2 mg) and NHS (5.5 mg) in DMSO (5.0 mL).After stirring the mixture for 4 h at room temperature in dark, DHA(-CONHNH_2_) (synthetized earlier) was added and stirred in the same manner overnight. The obtained crude product DHA-Cypate was then purified by silica gel column. The absorbance and fluorescence spectra of DHA-Cypate were recorded on a UV-vis spectrophotometer and an eight-channel optical fiber spectrofluorometer. The mass spectra were performed to verify the molecular weight of the probe [33].

### *In vitro* cell targeting assay and uptake mechanism study

#### Tumor targeting assay

Due to the lack of a near infrared light detector in our confocal fluorescence microscope, a visible fluorescent dye Rhodamine B (RhB) was labeled to DHA to evaluate its tumor-targeting efficacy at cellular level. The labeling process was conducted by a similar method used for the synthesis of DHA-Cypate described above. About 3×10^5^ MFC-7, HepG-2 tumor cells and normal liver cell L02 were seeded at the confocal petri dish and incubated at 37°C for 24 h. Subsequently, a 200-μL DHA-RhB DMSO solution (100 nmol/L) was added to the culture and incubated for 1 h. The non-conjugated dye (RhB) was used as the negative control and was treated with the same procedure. After washing three times with PBS, the cells were examined using laser confocal fluorescence microscope (LCFM).

#### Cellular uptake of DHA and its correlation with PE contents

Firstly, free DHA was incubated with the tumor cells (MCF-7, HepG-2) for 30 minutes, followed by the addition of DHA-RhB probe and incubation for another hour. The fluorescence was observed under Laser confocal microscopy. Secondly, the same dose of DHA and PE were simultaneously added in the same tumor cell lines in another group and incubated for 30 minutes, followed by the addition of DHA-RhB for continuous 1h to study the function of PE in the cellular uptake of DHA.

Thirdly, the PE contents in cell membrane were determined to study its correlation with cellular uptake. Because the direct determination of PE contents is complicated, the viscosity of cell membrane was measured to indirectly reflect the PE contents. Briefly, the tumor cells in logarithmic phase were incubated with DPH fluorescence solution for 30 minutes to allow enough DPH to insert into the cell membrane. The cells were washed again with DPS solution and suspended in 4ml PBS solution. The fluorescence intensities of I_VV_ and I_VH_ were detected by Fluorospectro-photometer to calculate the fluorescent polarization P, with excitation wavelength at 362nm and emission wavelength at 432nm. The P formula is described in the following:

P=(I_VV_-I_VH_)/(I_VV_+I_VH_),here,I_VV_ presents the fluorescence intensities when polarization and analysis of polarized plate are placed in a vertical position and I_VH_ is the fluorescence intensities when polarization of polarized plate is placed in a vertical position but analysis of polarized plate is in a horizontal position. The viscosity of cell membrane was calculated as: η=2P/(0.46-P)

Finally, the cell uptake of DHA was correlated with PE contents.

#### Cytotoxic assay

The human cell lines MCF-7 (breast cancer cells), HepG-2 (liver cancer cells) and L02 (liver normal cells) were purchased from American Type Culture Collection (ATCC; Manassas, VA, USA). Cells were cultured in a humidified atmosphere of 5% CO2 at 37°C in DMEM and RPMI1640 medium supplemented with 10% fetal bovine serum, 100 μg/mL penicillin and 100 μg/mL streptomycin.

DHA cytotoxicity was evaluated using cell viability assay. MCF-7 and HepG-2 cells were seeded in a 96-well plate (1×10^4^ cells/well). After cultivation for 24 h, DHA-Cypate (DMSO dissolve first,then added it into the cell culture medium)of different concentrations were added into the wells (n = 6) and incubated for 48 h. Then stock solution of MTT (20 μl; 5 mg/ml) was added into each well. After 4h incubation at 37 °C, the MTT solution was replaced with 150 μl DMSO in each well. The absorbance in each well was measured at 570 nm with a multi-well plate reader. Cell viability was calculated using the following formula: Cell viability = (Mean absorbance of test wells – Mean absorbance of medium control wells) / (Mean absorbance of untreated wells – Mean absorbance of medium control well) × 100%.

### Tumor-targeting ability of DHA-Cypate in tumor-bearing mice

#### Tumor model

Athymic nude mice (nu/nu, age 4–6 weeks and weight 18–22 g) were purchased from Charles River Laboratories (Shanghai, China). All animal experiments were carried out in compliance with the Animal Management Rules of the Ministry of Health of the People's Republic of China (document NO. 55, 2001) and the guidelines for the Care and Use of Laboratory Animals of China Pharmaceutical University.

In this study, MCF-7 and HepG-2 tumor models were used to investigate the *in vivo* targeting ability of the probe. Briefly, a suspension of ~5×10^6^ cancer cells (MCF-7 or HepG-2) in 100 μL phosphate buffered saline (PBS, 0.01 mol/L; pH=7.2) were subcutaneously injected into the axillary fossa of each mouse. When the tumors reached 0.4~0.6 cm in diameter, the tumor bearing mice were studied *in vivo* (n = 5 for each imaging probe or dye).

#### Tumor targeting ability assay

To evaluate the tumor-targeting capability of DHA-Cypate, 0.2 mL DHA-Cypate (2 nmol) (polyoxyethylene castor oil and absolute ethanol (1:1) were used as co-solvents to dissolve in the saline)was intravenously injected into MCF-7 and HepG-2 tumor-bearing mice, respectively, when the primary tumors reached a size of 0.4 to 0.6 cm in diameter. Near infrared fluorescence imaging of the tumor-bearing mice was acquired at designated time points of 0.5h, 2h, 4h, 6h, 8h, 12h, 24h.

Background images were taken for each mouse prior to probe injection. In addition, for quantifying the fluorescence of probe biodistributions in the mice body, the tumor/normal tissue ratio (T/N ratio) was analyzed and compared using the ROI function of the analysis. The subjected mice were sacrificed 24 hours post injection and the main organs were separated for NIR imaging to confirm the bio distribution of the probe.

To verify the mechanism of DHA for tumor targeting ability, free DHA and combination of free DHA with PE were respectively administrated into the tumor bearing mice in other different mice groups for comparison. The procedures were same as above mentioned. Data were expressed as a means ± SD (n =5).

#### Antitumor efficacy of DHA-GEM

To explore the potential application of DHA as a tumor targeting ligand, a novel antitumor drug, DHA-GEM, was constructed by conjugating the DHA with the anticancer drug GEM. The therapeutic efficacies of DHA-GEM were investigated *in vitro* and *in vivo*.

#### Synthesis and characterization of DHA-GEM

DHA-GEM was synthesized based on the literature [35], by conjugating N4-amino group of GEM with conjugated DHA. Briefly, DHA (0.54g), triethylamine (TEA, 280μl) were dissolved in a tetrahydrofuran (THF, 20mL) solution and then were added in a three-neck round-bottomed flask and cooled down to -10°C. Afterwards, ethylchlorocarbonate (0.2g) was added dropwise to the above THF solution mixture under Nitrogen atmosphere and was continuously stirred at −15°C for 30 min. Then gemcitabine hydrochloride (0.5g) and triethylamine (TEA, 280μL) were dissolved in the anhydrous dimethylformamide (DMF) solution. Finally, the DMF solution was added dropwise to the THF reaction mixture under stirring at the same temperature for 30 min. Later on, it was stirred for 72h at room temperature. After the reaction, the mixture was concentrated in vacuum. The crude product was purified to get the yellow solid product (0.42g, 45% yield) by using silica gel chromatography eluting gradually with 2% methanol in dichloromethane (0.42g, 45% yield). Q-TOF Micro Mass Spectrometer (Waters) was used to confirm the successful synthesis of DHA-GEM.

#### *In vitro* cytotoxicity evaluation

The cytotoxicity of DHA, gemcitabine and DHA-GEM were evaluated on MCF-7, HepG-2 and H22 (mouse hepatoma cancer)tumor cells. To study the broad spectrum of DHA-GEM, other five kinds of tumor cells [A549 (lung cancer), 7402 (liver cancer), MB-MDA-231 (breast cancer), 7901 (gastric cancer)] were used to investigate the cytotoxicity of gemcitabine and DHA-GEM.

#### *In vivo* antitumor efficacy of DHA-GEM

The human breast tumor cells MCF-7 and mouse hepatoma tumor cells H22 were subcutaneously injected into the upper right axillary fossa in the nude mice or Kunming mice (Charles River Laboratories) respectively. The H22 tumor cell implanted-Kunming mice were randomly assigned into 3 groups (n=10 per group). In 24 hours post-inoculation, saline solution (0.9%), gemcitabine solution (dissolved in saline, 40mg/kg equivalent to gemcitabine), and DHA-GEM solution (castor oil solution dissolved in diluted 1:1 with ethanol 50% v/v, 40mg/kg equivalent to gemcitabine) were administrated into the different group of mice on the days 1,4 and 7 via tail vein for the study of antitumor efficacy. Similarly, the MCF-7 bearing nude mice were also divided into 3 groups (n=6) and treated on the days 8,12,16,20 post-inoculation by using the same procedures as Kunming mice, with dose of 25mg/kg equivalent to gemcitabine. The therapeutic efficacies were assessed by measuring tumor volume and body weight every other day till the 9th day (Kunming mice-bearing H22 group)or 24th day (nude mice-bearing MCF-7 group).The tumor weight was measured in the last day at the time of sacrifice. The Kunming mice group survival rates were recorded.

### Histology examination

To further investigate the side effects of DHA-GEM on various organs of the treated mice, histological analysis of different organs was conducted by using the established technique.

### Statistical analysis

Data were expressed as mean ± SD. Statistical analysis was conducted by using Students t test with statistical significance assigned for P value less than 0.05.

## CONCLUSION

In our study we synthesized DHA-based NIR probes for the first time, and then showed the probes have a high *in vivo* and *in vitro* affinity to the tumor cells. We showed that PE had a close connection with tumor targeting mechanism of DHA. We have also synthesized a novel antitumor drug DHA-GEM. Our results showed that DHA-GEM had high efficacy and less toxicity than the free gemcitabine. This study proved that DHA, a naturally available nontoxic substance, is a good candidate for conjugation with the antitumor drugs to increase their efficacy and specificity. Furthermore, the fluorescent emission of DHA can be used for tumor diagnosis.

## SUPPLEMENTARY FIGURES


