# Supplementary material for: The targeting mechanism of DHA ligand and its conjugate with Gemcitabine for the enhanced tumor therapy
Source: Oncotarget. 2014 May 13;5(11):3622–35. doi: 10.18632/oncotarget.1969 (PMC4116508; doi:10.18632/oncotarget.1969)
Supplement: Supplementary file 1 [file oncotarget-05-3622-s001.pdf]

The targeting mechanism of DHA ligand and its conjugate with Gemcitabine for the enhanced tumor therapy

Supplementary Material

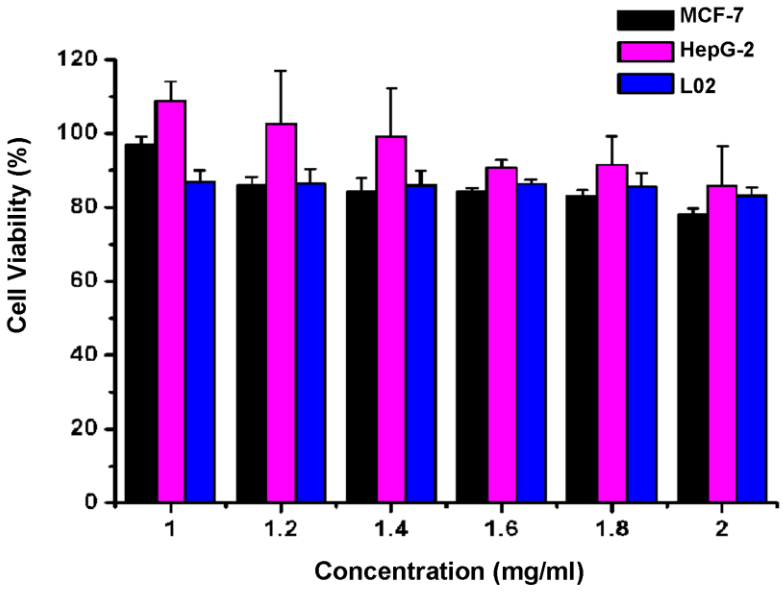

Supplementary Figure 1: Cytotoxicity of DHA-Cypate probe. Cell viability of MCF-7 cells, HepG-2 cells and L02 cells incubated with different concentration DHA-Cypate probe. Data are given as mean ± SD (n=5)

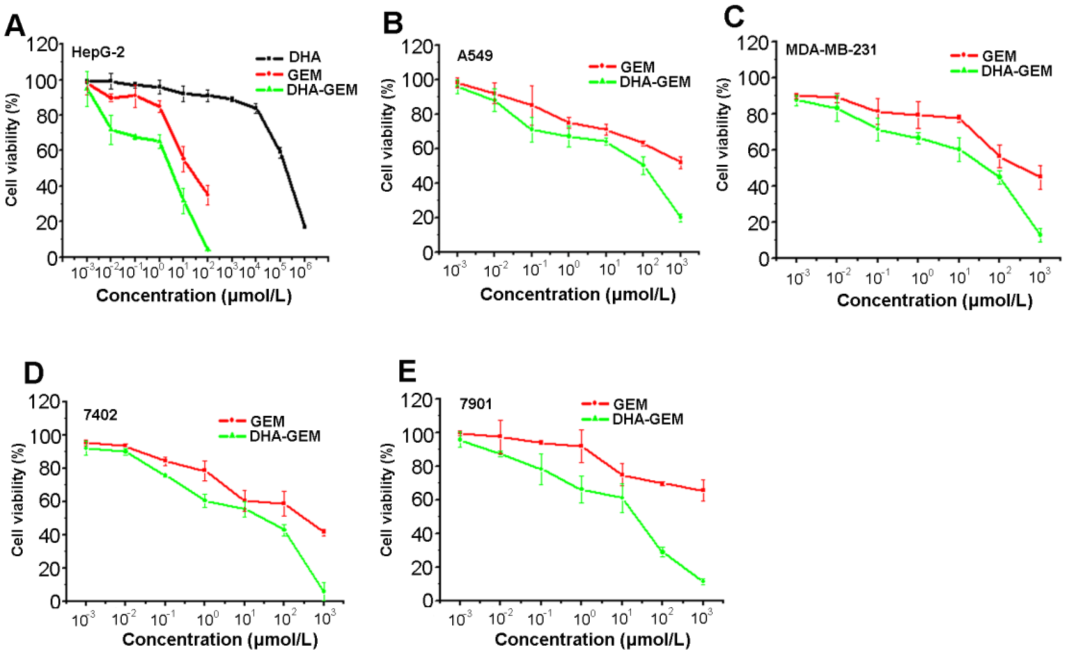

Supplementary Figure 2: *In vitro* antitumor efficacy of DHA-GEM on (A) HepG-2 cells incubated with DHA, free gemcitabine and DHA-GEM.(B)A549,（C）MDA-MB-231, (D) 7402, (E) 7901 incubated with free gemcitabine and DHA-GEM. Data are given as mean ± SD (n=5)
